# Three-Dimensional Bioprinting Techniques in Skin Regeneration: Current Insights and Future Perspectives

**DOI:** 10.3390/life15050787

**Published:** 2025-05-15

**Authors:** Anna Barbara Di Stefano, Valentina Urrata, Kim Schilders, Mara Franza, Simona Di Leo, Francesco Moschella, Adriana Cordova, Francesca Toia

**Affiliations:** 1 BIOPLAST-Laboratory of BIOlogy and Regenerative Medicine-PLASTic Surgery, Plastic and Reconstructive Surgery Section, Department of Precision Medicine in Medical, Surgical and Critical Care, University of Palermo, 90127 Palermo, Italy; annabarbara.distefano@unipa.it (A.B.D.S.); valy2707@gmail.com (V.U.); simona.dileo@unipa.it (S.D.L.); francesco.moschella@unipa.it (F.M.); adriana.cordova@unipa.it (A.C.); francesca.toia@unipa.it (F.T.); 2 Alliance of Dutch Burn Care, Burn Research Lab, 1941 AJ Beverwijk, The Netherlands; 3 Plastic and Reconstructive Surgery Section, Department of Precision Medicine in Medical, Surgical and Critical Care, University of Palermo, 90127 Palermo, Italy; mara.franza@unipa.it; 4 Section of Plastic and Reconstructive Surgery, Department of DAI chirurgico, Azienda Policlinico Paolo Giaccone, 90127 Palermo, Italy

**Keywords:** 3D bioprinting, bioinks, scaffolds, skin damage, extracellular vesicles

## Abstract

Skin is composed of three layers: the epidermis, dermis, and hypodermis. It is enriched with skin appendages, including hair follicles, sweat glands, and sebaceous glands, which play essential roles in regulating fluid exchange, controlling body temperature, and providing protection against pathogens. Currently, skin regeneration treatments rely on transplantations. However, this approach has several disadvantages, including hemostasis at the recipient site, limitations in donor area closure, increased graft contraction, and hypertrophic scarring. Recent advancements in three-dimensional (3D) bioprinting technologies have enabled the fabrication of structures that closely mimic native tissues, with the aim of enhancing tissue regeneration. Bioprinting offers several advantages, such as high reproducibility, precision, and the ability to create complex geometries. The most promising bioinks combine excellent biocompatibility and biodegradability, with mechanical and rheological stability. This review highlights the most recent and innovative studies on 3D-printed bioinks in the field of skin tissue engineering. In particular, considering the growing interest in the regenerative potential of exosomes, we discuss cutting-edge research involving exosome-loaded bioinks and their potential to support skin regeneration and repair.

## 1. Introduction

### 1.1. Skin Anatomy and Physiology

Skin is a complex organ that covers a surface area of 1.8 m^2^ on the human body [1]. It is divided into three layers: the epidermis, dermis, and hypodermis. The epidermis comprises keratinocytes (KCs), melanocytes, Langerhans, and Merkel cells. Maintaining integrity is important because of its protective function. The dermis is the intermediate layer composed of connective tissue, lymphatic vessels, blood vessels, hair follicles, and the sebaceous and sweat glands. It is mainly formed by fibroblasts (FBs) and type I and III collagen. The hypodermis is the deeper layer of the skin and consists of connective tissue and fat, together with sensory neurons and blood vessels [2,3]. Similar to all other organs, skin can be subjected to damage. The main concern with extended damage is that the wound site may undergo abnormal tissue regeneration, leading to scar formation. This compromises its physiological function and aesthetic appearance [4]. Moreover, the inability to regenerate skin appendages after extended damage remains problematic [5,6]. These appendages, are important because they regulate excretion, perspiration, and thermoregulation [7]. Hair follicles, widely distributed in several regions of the body, act as the first line of defense against external environmental stimuli and contaminants. They are also involved in thermoregulation and wound healing [8]. After damage, epithelial stem cells from the hair follicle migrate towards the injured site and differentiate into epidermal cells, thereby leading to re-epithelialization of the wound area [9,10]. An important antimicrobial function is performed by sebaceous glands, which secrete a slightly acidic sebum on the surface of the skin to protect it from viruses, bacteria, and other contaminants. Sweat glands play important roles in regulating body temperature and maintaining homeostasis in body fluids [11]. Skin appendages develop during the embryonic phase and maintain several populations of stem cells that are capable of regeneration. In addition, these stem cells can participate in skin wound healing after injury. However, in cases of extensive damage, the regenerative potential may be severely compromised [12].

### 1.2. Skin Injuries

Skin injuries are commonly classified as acute or chronic, depending on the healing dynamics and duration [13]. Acute wounds lead to the recovery of anatomical and functional integrity and are articulated in four phases: hemostasis, inflammation, proliferation, and remodeling. In the last phase, activated FBs, called myofibroblasts, regulate the contraction and maturation of the granulation tissue. The synthesis of extracellular matrix (ECM) is reduced, collagen type III is replaced by collagen type I, and elastin is secreted. Chronic wounds lead to tissue fibrosis and ulcers due to prolonged inflammatory and proliferative phases, which do not allow a common healing procedure [14,15]. Although several factors, such as age, underlying diseases, and drug therapy, may affect the wound healing process, several studies suggest a direct correlation between the extent of the injury (which is associated with higher inflammation levels) and the amount of scar tissue. Deep wounds also lack secretion of elastin fibers, leading to the formation of non-elastic skin [15,16].

### 1.3. Skin Treatments

The current treatments for skin regeneration include transplantation, dermal substitutes, and wound sprays. Skin transplantation involves the transfer of a graft from the donor to the recipient site [17]. It is important that these grafts carry two layers: the epidermis and the dermis. The presence of the dermis is essential because the epidermis is avascular, and healing can only occur through the connection between the host bed and graft vasculature. The dermis can be partially included (split-thickness skin grafting) or completely included (full-thickness skin grafting). Both these methods require a well-vascularized recipient site and should be free of bacterial contamination and devitalized tissue. A common disadvantage of skin transplantation is hemostasis at the recipient site, because the graft can fail due to hematoma formation. Another important limitation is the extension of the piece taken from the donor in the extended autologous full-thickness skin grafts. In autologous split-thickness skin grafting, large available donor areas and a better graft ‘take’ can be obtained, but some significant disadvantages are the increased graft contraction and the hypertrophic scarring. The survival of skin autografts is permanent, whereas the survival of skin allografts is only temporary until rejection occurs [18,19,20,21,22]. Dermal substitutes and wound sprays can also be used in combination with autologous skin transplantations. Dermal substitutes are biomatrices that mimic the properties of the ECM and replace the dermis. As the dermis cannot regenerate after injury, dermal substitutes must promote new tissue growth and enhance wound healing. Dermal substitutes can be applied underneath autologous skin grafts to improve the wound healing process [23] and are very useful in the case of deep wounds or bone or tendon exposure [24]. Wound sprays are cellular or acellular sprays that can be easily applied homogeneously to the damaged site. When cellular sprays are used, it is important to choose an appropriate quantity of cells; instead, acellular sprays are usually composed of hydrogels that layer over the wound, covering it and protecting it from infection and fluid loss. They can also be used on extended wound surfaces and can perform antibacterial, pain reduction, and hemostatic functions, as in the case of fibrin hydrogel as a skin spray [25,26]

### 1.4. New Approaches for Skin Regeneration

Three-dimensional (3D) bioprinting is a relatively new technique developed to generate structures that simulate native conditions. It allows the generation of scaffolds made with bioinks that should have three main characteristics: the ability to be 3D-bioprinted, biocompatibility with living cells, and biodegradability. Biocompatibility and biodegradability are fundamental aspects in the design of an effective bioink. The produced scaffold must support cell growth without triggering adverse responses, gradually degrade to enable new tissue formation, and possess adequate mechanical properties to ensure stability and flexibility. Bioprinting allows the creation of complex geometries and offers several key advantages, including repeatability and precision. Moreover, 3D-bioprinted structures exhibit regular porosity, which regulates cell attachment, growth, nutrient supply, and waste product removal [27,28]. Porosity, in particular, plays an essential role in the final structure, because it facilitates oxygen and nutrient diffusion while limiting the accumulation of exudates, promoting faster healing. However, pore size could represent a limitation, since excessive porosity may reduce the antibacterial ability of the material and hinder scaffold re-epithelialization. The main goal of 3D bioprinting in the field of cell biology is to generate tissues and organs that can be implanted into patients to replace damaged tissues [29,30]. Three-dimensional bioprinting has already been applied in the generation and/or transplantation of cardiac tissues [31,32], skeletal muscle [33], trachea [34], and the liver [35]. This technique could also be used for skin regeneration, thereby solving the significant limitations of skin transplantation. Several natural bioinks have been developed, and this innovative technique has been used to generate scaffolds, together with cells, or subsequently colonized, with the aim of regenerating full-thickness skin after damage. Bioinks are composed of hydrogels with or without cells such as FBs, KCs, endothelial cells, and adipose- or bone-marrow-derived stem cells [36].

In addition, extracellular vesicles (EVs) have become a major focus of scientific interest due to their ability to deliver information to cells. They can be divided into exosomes (30–150 nm), microvesicles (100–1000 nm), and apoptotic bodies (50–5000 nm). Among the two most widely used types of EVs, exosomes are round-shaped vesicles derived from multivesicular bodies (MVBs) that originate from early endosomes. MVBs contain intraluminal vesicles (ILVs) that are produced through the inward budding of endosomal membranes. MVBs have two possible fates: they can either merge with lysosomes for degradation or they can fuse with the plasma membrane and release their EVs content outside the cells. The ILVs released in this manner are referred to as exosomes [37]. Microvesicles with irregular shapes are derived by the plasma membrane shedding and are directly released into the extracellular space. Their surfaces are characterized by membrane components typical of the cell of origin [38]. Both are enriched in small and long non-coding RNAs, mRNAs, lipids, and proteins that convey specific information to the recipient cells [39,40].

This narrative review summarizes recent studies on the use of three-dimensional scaffolds generated through 3D bioprinting of innovative bioinks, applied to skin regeneration and the development of skin-equivalent models, both in vitro and in vivo. The main contribution of this review is to highlight emerging materials and advanced printing techniques used in regenerative medicine, with a particular focus on the role of exosomes as bioactive components of bioinks, the modulation of their release at the injury site, and the use of portable biomedical devices for effective and localized application.

## 2. Three-Dimensional Printing in Skin Regeneration

One of the main challenges in regenerative medicine is treating skin wounds from various conditions that cause significant tissue loss. In recent years, a potential solution has emerged through 3D printing. The bioinks used are biocompatible and non-toxic formulations that promote cell proliferation, migration, and differentiation, thereby contributing to the repair and regeneration of damaged tissue.

Recently, research has focused on adding bioactive extracts to bioinks to enhance their antimicrobial and antioxidant properties. In this context, the methanolic extract of *Satureja cuneifolia* (SC), a plant with recognized antidiabetic and antimicrobial properties, was integrated into scaffolds based on sodium alginate and polyethylene glycol. This approach, while offering documented antimicrobial properties against Gram-positive bacteria such as *Staphylococcus aureus*, proves to be less effective against Gram-negative strains like *Escherichia coli*, likely due to the limited ability of essential oils to penetrate the hydrophobic bacterial membranes [41,42]

This combination has shown promising potential in treating diabetic ulcers by reducing infections and accelerating tissue regeneration [43]. Although increasing the concentration of SC to enhance its antimicrobial effect may appear attractive, it poses a risk of toxicity. Carvacrol, one of the main bioactive compounds of SC, is known for its dose-dependent cytotoxic effect [44], and higher concentrations could exacerbate this issue. Furthermore, despite documented antifungal activity against *Candida albicans* and *Saccharomyces cerevisiae*, as well as antibacterial activity against *Salmonella typhimurium*, further investigations are needed to ensure a safer and more controlled application of the extract. 

In contrast, the use of silver as an antimicrobial agent in 3D-printed scaffolds appears to overcome some of the limitations associated with natural additives. In particular, a recent study demonstrated that a bilayer substrate—comprising an upper layer of silver-loaded cryogelatin and a lower layer containing PDGF-BB—can successfully combine both antimicrobial and regenerative effects within a single platform. Silver exhibited strong antibacterial activity against clinically relevant strains, including *P. aeruginosa*, *S. aureus*, and *E. coli*, without showing significant cytotoxicity toward KCs, FBs, and immune cells. Although it did not directly enhance the regenerative potential compared to PDGF-BB alone, its presence contributes to the prevention of infection, which remains a critical barrier in tissue regeneration [45].

Additional research has focused on designing asymmetric surfaces with wettability properties for skin regeneration. An innovative composite dressing was developed by combining electrospinning and bioinspired micropatterning, resulting in an outer hydrophobic layer of PCL nanofibers, which prevents bacterial adhesion and protects the wound from external agents, and an inner hydrophilic layer of gelatin nanofibers incorporated with pioglitazone (Gel-Pio), which stimulates cell proliferation, migration, and angiogenesis. In vitro tests confirmed biocompatibility with human FB and endothelial cells, showing a significant increase in cell proliferation and angiogenesis compared to controls. The dressing’s effectiveness was tested in vivo on murine models of diabetic wounds, demonstrating faster wound healing compared to conventional treatments such as Tegaderm and gauze. Treated animals showed more effective tissue regeneration, with greater collagen deposition and the formation of new blood vessels. The analysis of inflammatory markers revealed a significant reduction in MMP-9, IL-1β, and IL-6 expression, accompanied by increased cell proliferation (Ki67) and angiogenesis, as demonstrated by VEGF and CD31 levels. The outer hydrophobic layer was made of polyester-caprolactone (PCL) nanofibers deposited via electrospinning on nylon meshes with 40 µm and 80 µm pores, while the inner hydrophilic layer consisted of gelatin nanofibers encapsulated with pioglitazone and stabilized through crosslinking with genipin. The analysis showed that the dressing with PCL deposited on 40 µm meshes (PCL40) provided the best results in terms of protection and healing. Compared to other tested configurations, PCL40 demonstrated greater hydrophobicity, significantly reducing bacterial adhesion, especially against Escherichia coli and Pseudomonas aeruginosa [46].

## 3. Bioinks

In the field of skin regeneration, 3D printing bioinks are playing an increasingly important role. These materials, designed to mimic the natural extracellular matrix (ECM) of tissues, promote tissue regeneration by stimulating cellular activity and are applied in the treatment of skin wounds caused by various pathologies [47]. Bioinks are primarily composed of polymers, which form their fundamental structure. Among the most commonly used polymers in 3D printing, hydrogels are particularly advantageous due to their crosslinked structure, which allows them to absorb and retain large amounts of water [48]. This characteristic enhances the material’s stability and consistency, making it ideal for tissue engineering applications. Hydrogels can be derived from natural sources, such as plant, animal, or human tissues, or synthesized through chemical processes. In many cases, they are combined to create hybrid formulations that improve the overall properties of the material.

A significant example is provided by a scientific study conducted by Muscolino et al., in which the combination of κ-carrageenan (kC) and polyvinyl alcohol (PVA) demonstrated unique properties, making the material suitable for facial cartilage reconstruction through 3D bioprinting. κ-Carrageenan is a thermoresponsive biopolymer that remains in liquid form at temperatures above 50–60 °C and rapidly undergoes physical gelation upon cooling. This property enables the creation of a moist microenvironment, which is optimal for cell growth and tissue regeneration [49]. The addition of PVA further enhances the material’s properties by reducing viscosity and lowering the gelation temperature, making the system more workable and adaptable to bioprinting processes. Additionally, PVA improves the mechanical stability of the hydrogel by increasing its strength and flexibility while preventing excessive brittleness. Another key advantage of PVA is its ability to modulate the material’s porosity, promoting nutrient diffusion and cellular infiltration—essential factors for the development of functional scaffolds. This combination results in a material that is highly moldable and stable over time, which are essential characteristics for 3D printing. Furthermore, the ability to form hydrogels at biologically compatible temperatures makes its use particularly promising in regenerative medicine.

### 3.1. Bioinks Without Cells

There are many studies in which innovative bioinks were developed without incorporating cells, and each of them has important and original characteristics. In one study, patches of approximately 15 mm in diameter, made of 3D-printed thin filaments of 15% GelMA (Gelatinmethacryloyl) and 5% HAMA (hyaluronic acid methacryloyl), were generated. After bioprinting, the patches were conjugated with 100 mg of QHREDGS, a soluble angiopoeitin-1 (Ang-1)-derived peptide with proangiogenic functions. An in vivo rat skin wound model showed that 3D-bioprinted GelMA/HAMA-QHREDGS patch application led to quick and almost complete closure of the wound with the generation of thick and vascularized skin, together with ECM neo-formation, as proven by collagen deposition at the wound site. Simultaneously, the expression levels of pro-inflammatory interleukin-6 (IL-6) and tumor necrosis factor-α (TNF-α) were also increased [50]. Additionally, the properties of placenta-derived ECM were tested to generate printable bioinks to mimic the skin. This ECM was chosen for its properties, including the absence of cytotoxicity, its ability to prevent immune responses, and its easier availability. ECM with a concentration of 5% was mixed with 6% gelatin and 6% sodium alginate to form ECM-Alg-gel bioink. In vivo full-thickness dorsal wound mouse models were generated, and 5% ECM-Alg-Gel scaffolds were implanted. A wound healing percentage of 86.66% was obtained after 21 days, confirmed by overexpression of TGFβ1, Col1a, and Col3a. Moreover, the regenerated epidermis showed a cellular structure very similar to native skin and contained appendages, such as hair follicles. Overexpression of pro-angiogenic genes, such as bFGF, VEGFA, and VEGFR, could be related to a high number of new blood vessels and new collagen synthesis. Moreover, an ex vivo CAM assay on chick embryos showed that scaffolds induced angiogenesis [51]. In conclusion, this study demonstrated that 5% ECM-Alg-Gel scaffolds significantly accelerated wound healing in chronic deep wounds, showing a reduced rejection rate compared to ECM-free scaffolds and untreated groups. The innovative combination of placenta-derived ECM with Alg/Gel not only facilitated rapid integration with host tissues, but also promoted significant neovascularization and re-epithelialization. The SDS/Triton decellularization protocol effectively preserved ECM components while removing cellular debris. These results suggest that incorporating ECM into 3D-printed biomaterials creates a favorable environment for tissue regeneration, enhancing biological responses compared to ECM-free scaffolds. Another study developed 3D-printed scaffolds via syringe extrusion (SSE-3D), using xanthan gum (XG) and guar gum (GG) mixtures for wound healing applications. The 12% *w/v* XG/GG mixture with a 50:50 ratio exhibited optimal viscoelastic properties, enabling the printing of porous scaffolds with high shape fidelity. The addition of organic acids (citric, succinic, and tartaric) improved the mechanical properties and stability without affecting printability, making the scaffolds suitable for implantable applications. The hybrid bioink demonstrated the ability to rapidly form stable hydrogels, without requiring external stimuli such as temperature or pH shifts, or the presence of crosslinking ions typically necessary for gelation in other polysaccharide-based systems like gelatin, chitosan, carrageenan, or alginate. This intrinsic self-gelling capability confers a notable advantage in terms of printability, facilitating streamlined processing, improved reproducibility, and enhanced suitability for extrusion-based bioprinting applications. Structurally and mechanically, crosslinked scaffolds maintained their shape in PBS for at least seven days, while non-crosslinked ones showed swelling and loss of integrity. The S–SA scaffold demonstrated the best compressive strength, with sponge-like elastic behavior, capable of absorbing and releasing fluids without losing stability. SEM analysis revealed uniform microporosity, promoting cell migration and nutrient transport, crucial for tissue regeneration. Biological evaluations confirmed high cytocompatibility with mesenchymal stem cells (hMSCs) without toxic effects from crosslinking. The in ovo CAM test showed pro-angiogenic activity for S–SA and S–TA, with the formation of blood vessels and microcapillaries around the scaffolds, suggesting good tissue integration. S–SA’s ability to inhibit collagenase activity highlights its potential in treating deep chronic wounds, where excessive collagen degradation impedes healing. Moreover, the scaffolds bound divalent ions like Ca^2+^ and Zn^2+^, essential for tissue regeneration and regulation of metalloproteases [52]. We describe the studies on the bioinks without cells in Table 1.

### 3.2. Bioinks with Cells

Several studies, discussed in the following subsections, used cells bioprinted together with materials, or cells seeded on top of them, to generate innovative bioinks. The most commonly used cell types are FBs, KCs, spheroids of dermal papilla cells, endothelial cells, and adipose-derived stem cells (ADSCs). A comprehensive overview of all cell-loaded bioinks is provided in Table 2.

#### 3.2.1. Fibroblasts

FBs have been used as a single-cell line added to the bioink mixture. Cells were loaded in different 3D-bioprinted scaffolds: a crosslinked hydrogel made of 4% GelMA (Gelatin Methacryloyl) and 8% SilMA (methacrylated silk fibroin), named GelMA-SilMA hydrogel, and carbohydrazide-crosslinked (polyethylene oxide-co- Chitosan-co- poly(methylmethacrylic-acid) called PEO-CS-PMMA, loaded with nicotinamide (NA). In the GelMA-SilMA hydrogel bioink, mice-derived FBs were mixed at a concentration of 20 × 10^6^ cells/mL, and a patch with a porous network 100 µm in size was generated. Instead, PEO-CS-PMMA was bioprinted using two different cartridges: one loaded with bioink and the other loaded with an NA-human dermal fibroblast (HDFs) mixture. A porous structure with pore dimensions of 2–10 μm was generated. Both bioinks were tested in vivo and in vitro. The GelMA-SilMA hydrogel showed the capacity for wound healing and new extracellular matrix (ECM) synthesis, together with the ability to regenerate a thick and vascularized epidermis, while PEO-CS-PMMA, loaded with NA-HDF, showed good structural properties, such as good viscosity, high elasticity, high tensile strength, and self-healing properties, owing to its homogeneous and organized PEO dispersion inside the scaffold [53,54]. Another study used an innovative bioprinting approach called ‘prestress bioprinting’ to bioprint cylindrical hydrogel filaments subjected to static tensile stress force during the process. This enabled the induction of cell-oriented growth. The cells were loaded into a Teflon tubing mold and used as bioink. Gelatin and microbial transglutaminase (mTG) were used to slow the crosslinking of the bioink. Stretched human skin fibroblast (HFF-1)-laden scaffolds decreased the dimensions of full-thickness skin wounds in vivo immunodeficient mice. Moreover, the granulation tissue was thick, and FBs expressed higher levels of PCNA, demonstrating their high proliferation activity and high VEGF levels, promoting angiogenic phenomena [55]. Another study explored the development and evaluation of a hybrid bioink composed of Gelatin Methacryloyl (GelMA) and a skin-derived extracellular matrix (SdECM), aimed at enhancing the printability and biofunctionality of 3D-bioprinted scaffolds for skin tissue engineering. The authors analyzed the rheological properties of the GelMA/SdECM bioink, observing the material’s behavior at different temperatures and with the use of UV radiation for material curing. The results showed that the hybrid bioink, compared to GelMA alone, exhibited higher viscosity, suggesting that SdECM contributed to improving the physical properties of the bioink, ensuring greater stability during printing. The bioinks examined displayed a “shear thinning” behavior, reducing viscosity under shear forces, thereby preserving the viability of encapsulated FBs during extrusion. In terms of cell viability, the hybrid bioink showed improved cell spreading and proliferation compared to GelMA alone, with a cell viability rate of 97.6% for the GelMA/SdECM hybrid, compared to 96.2% for GelMA alone. The FBs continued to proliferate during the 7-day test, suggesting that the hybrid bioink supports cell growth and preserves good cell morphology. Gene expression analysis revealed that the hybrid bioink increased the expression of typical skin markers, such as type 1 collagen and fibroblast growth factor, compared to pure GelMA. These results highlight how the integration of SdECM into the bioink not only improves its physical properties and printability but also enhances its biological functionality, making it a promising candidate for advanced applications in skin tissue engineering [56].

#### 3.2.2. Fibroblasts and Keratinocytes Combined with Spheroids of Dermal Papilla Cells Spheroid

FBs have also been combined with other cell lineages to generate innovative bioinks. In the GelMA-rhCol3-3.2 hydrogel, made of 3.2% of a recombinant type III collagen (rhCol3) and 7.5% GelMA, human dermal FBs (HDFs) were added before bioprinting at a concentration of 1 × 10^6^/mL while, on the top of the crosslinked structure, 2 × 10^5^ cells/cm2 of human epidermal KCs (HaCaTs) were seeded. In vivo scaffold injection in full-thickness excisional rat wound models, followed by in situ UV-crosslinking to gelify the ink, showed a wound closure of 94%, together with the formation of new hair follicles and new extracellular matrix deposition [57]. Similarly, human FBs were printed together with a crosslinked hydrogel made of guanidinylated/PEGylated chitosan (GPCS) at a concentration of 5%, mixed with gelatin at a concentration of 3%, and type I collagen from rat tails at a concentration of 0.2%. After 3D bioprinting, 10^7^ cells/mL of HaCaT cells were seeded on top. In vitro analysis showed that KCs were able to proliferate and organize themselves into several epidermal layers, thereby mimicking full-thickness skin [58].Normal HDFs (NHDFs) at a concentration of 5 × 10^6^ cells/mL were used in combination with 100–150 hair follicle dermal papilla cell (HFDPC) spheroids. Both were incorporated into a gelatin methacryloyl/hyaluronic acid methacryloyl (GelMA/HAMA) bioink to generate dermal and epidermal layers. The mixture was 3D-bioprinted, generating a porous structure with a pore dimension of 118.40 ± 12.32 μm. After bioprinting, an additional layer of 10^6^ cells/mL NHDF-laden dermis was 3D-printed and, on top of the dermal structure, human immortalized keratinocyte (HaCaT) cells (10^5^ cells/mL) were seeded to generate the epidermis. To generate the lower dermis, a lattice cuboid was synthesized to provide oxygen and nutrients to the cells. In vitro analysis demonstrated that the structure was adapted to maintain high cell viability for both HaCaT and NHDF cells, and that it recreated the physiological distribution of cells into the native skin, together with its ECM composition. Moreover, HaCaT cells surrounding HFDPC spheroids generated a complex that organized into a hair-follicle-like unit (HFU) [59].

Other studies also loaded HaCaT cells into the bioink and seeded them on top of the 3D-printed structure. HDFs (4 × 10^6^ cells/mL) and HaCaT cells (6 × 10^6^ cells/mL) were separately loaded into the COL-ALG-20 bioink to mimic the dermis and epidermis, respectively. The COL-ALG-20 bioink was composed of semi-cross-linked alginate (ALG) mixed with 20 mg/mL marine collagen from basa fish skin. Marine collagen was chosen because it is easy to extract and has high anti-microbial activity. The scaffold had a circular shape, with a pore size of 83 ± 27 µm. In vitro studies have shown that cells maintain their viability inside these materials, and ECM components such as collagen types I and III, elastin, fibronectin, and CK6 are highly expressed [60]. In another study, hydrogel-based bioinks composed of two polysaccharides, Nanofibrillated Cellulose and Gellan Gum (NFC/GG), were developed with a ratio of 60:40. NFC exhibited good mechanical properties, whereas GG allowed the hydrogel to maintain its shape. The NFC/GG bioink was loaded with HaCaT cells at a concentration of 3 × 10^6^ cells mL^−1^ before printing. In vitro experiments showed that cells inside the two-polysaccharide-based hydrogel maintained high viability for seven days [61]. Gelatin-assisted extrusion bioprinting was used to print sacrificial gelatin bioink on a 3D-printed supporting structure made of a synthetic polymer. First, the porous membrane was seeded with a 3D dermis containing HDFs to simulate a dermal equivalent, and then human epidermal keratinocyte (HEK)-laden gelatin bioink was extracted at a cell concentration of 1 × 10^6^ cells/mL. The bioink was completely dissolved after 3 h of incubation at 37 °C, providing a suitable support for cell adhesion. In vitro analysis showed upregulation of epidermal markers for early differentiation, such as loricrin (LOR), involucrin (IVL), and keratin 1 (KRT1), and the ability of HEKs to differentiate from the lower to the upper layer of the skin by forming the stratum basale, spinosum, granulosum, lucidum, and corneum. Their layering in a native-like epidermis was demonstrated by the expression of high levels of tight junction proteins, such as claudin-1, zonula occludens-1 (ZO-1), and occludin [62].

#### 3.2.3. Fibroblasts, Keratinocytes and Endothelial Cells

Innovative studies have also integrated HUVECs into bioinks to improve skin vascularization during full-thickness regeneration. Strontium silicate (SS) microcylinders with a uniform morphology of hexagonal prisms and small size (15 μm) were synthesized and then integrated into an ink, called the GAM matrix, to enhance printability. SS was used to improve the angiogenic activity of the scaffolds. GAM matrix was composed of Gellan Gum (GG) hydrogel with a concentration of 2.8% and two high-viscosity materials such as sodium alginate (AG) with a concentration of 1.6%, and methyl cellulose (MC) at a concentration of 2.8%. HUVECs were bioprinted using the ‘cell writing’ technique to seed them on the lower part of the scaffold and HDFs on the upper. In vivo 15 mm full-thickness mice models showed a full skin regeneration of acute and chronic wounds when the Co-2SS-GAM scaffold (cells in 2% SS mixed with GAM) was applied. The regenerated skin was composed of dermal structure rich in collagen fibers. Moreover, complete epithelialization and hair follicle morphogenesis were observed. Blood vessel formation inside the dermis has been previously demonstrated [63]. In addition, an innovative bioink composed of a microfragmented adipose extracellular matrix (mFAECM), derived from adipose tissue digestion, was mixed with gelatin methacryloyl (GelMA) and hyaluronic acid methacryloyl (HAMA). mFAECM is rich in collagen and sulfated glycosaminoglycans and is mixed with GelMA and HAMA because of its good properties for bioprinting and adaptation for cell growth and survival. A scaffold composed of a HUVEC layer, two fibroblast layers, and a HaCaT layer was generated. An in vivo full-thickness excisional skin defect mouse model was generated, and the applied construct was able to regenerate the dermal structure by stimulating contraction of new tissue inside the wound, together with new vascularization and type III collagen deposition, thereby avoiding scar formation. The basic structures of implants are maintained throughout wound healing and gradually metabolized [64].

#### 3.2.4. Adipose- and Bone-Marrow-Derived Stem Cells

Adipose-derived stem cells have also been used to generate biocompatible skin substitutes, and in three recent studies, they were combined with a decellularized extracellular matrix as a bioink component. Human ADSCs at a concentration of 1 × 10^7^ cells/mL were added to a bioink composed of 1.125% human adipose tissue decellularized ECM (adECM), 7.5% GelMA, and 1% methacrylated hyaluronic acid (HAMA) at a 1:9 ratio. The adECM-GelMA-HAMA scaffold was bioprinted with a circular shape and with pore diameters of 131.39 ± 6.88 μm. The in vivo wound healing assay on a full-thickness injury nude mouse model proved that adECM-GelMA-HAMA ADSC-laden scaffolds led to wound closure, as confirmed by neovessel formation and collagen deposition [65]. Subsequently, the same research group implanted an adECM-GelMA-HAMA-crosslinked scaffold with a pore size of 73 ± 18 μm and cylindrical shape in a full-thickness skin wound mouse model. The results showed that after 14 days, the mice achieved complete wound healing with the formation of complete skin structures and only a small amount of scar tissue. In addition, a rich amount of collagen and moderate infiltration of inflammatory cells have been reported [66]. By using decellularized and solubilized porcine skin tissue that maintained the typical characteristics of skin ECM (S-dECM), a full-thickness 3D human skin model was bioprinted to generate a skin patch. Before bioprinting, it was loaded with 2.5 × 10^5^ endothelial progenitor cells (EPCs)/mL and 2.5 × 10^5^ ASCs/mL of the skin-derived bioink. A 10 mm dorsal wound mouse model was generated, and the bioprinted patch was implanted in the wound site for 21 days. The ASCs and EPC-printed skin patch showed a highly superior wound healing rate over 21 days compared to the control groups, and the dECM-ASC-EPC construct led to re-epithelialization and neovascularization, together with an improved blood flow at the wound site [67]. In recent years, numerous studies have demonstrated that the use of bone-marrow-derived mesenchymal stem cells (BM-MSCs) can promote effective wound healing. Among the most promising strategies for the treatment of difficult wounds, the use of 3D PLC-based scaffolds stands out. These scaffolds are enriched with 0.5% F-127 and immersed in a 0.5% gelatin solution, into which BM-MSCs are incorporated at the time of printing, actively stimulating tissue regeneration. The developed material not only promotes granulation tissue formation, angiogenesis, and collagen deposition but also induces macrophage polarization toward the M2 phenotype, enhancing the expression of anti-inflammatory cytokines such as IL-4 and IL-10, which are essential for inflammation resolution and the healing process. This mechanism helps create a pro-regenerative microenvironment, optimizing the healing process and offering an innovative alternative to current therapeutic solutions. For the first time, the possibility of creating customized structures capable of perfectly adapting to the size, depth, and morphology of diabetic wounds is highlighted. Compared to commercially available dressings for the treatment of diabetic foot ulcers (DFUs), these innovative scaffolds feature a unique structure composed of porous nanofibers with both radial and vertical orientation, potentially enhancing therapeutic efficacy [68].

## 4. EVs in Skin Regeneration

EVs are increasingly utilized in biomedical research and are commonly derived from various cell lines. In the context of skin regeneration, mesenchymal stem cells (MSCs) are widely employed due to their ability to secrete a broad range of bioactive molecules, including EVs, cytokines, chemokines, growth factors, and interleukins. These vesicles mediate intercellular communication by transmitting signals to target cells, thereby modulating cellular behavior and contributing to immune regulation. Moreover, they modulate neutrophil activity by inhibiting complement activation through CD59 and reducing prolonged inflammatory responses, thus preventing the formation of hypertrophic scars. They also promote the transition of macrophages from the M1 to M2 phenotype, enhancing the secretion of anti-inflammatory cytokines and growth factors, thereby facilitating tissue repair.

MSC-EVs (Mesenchimal Stem Cell Extracellular Vesicles) suppress the proliferation of pro-inflammatory T lymphocytes (Th1, Tc1) and promote the expansion of regulatory T lymphocytes (Tregs), further contributing to inflammation reduction. Further, they stimulate fibroblast and keratinocyte proliferation, collagen deposition, and neovascularization through the activation of various signaling pathways, including PI3K/AKT, Wnt/β-catenin, and Mir-93-3p/APAF1.

In particular, EVs derived from adipose-derived stem cells are distinguished by their ability to promote wound healing without scar formation by regulating the secretion of extracellular matrix components, inhibiting excessive fibroblast proliferation, and reducing inflammation [69].

In a murine model of chronic diabetic wounds, the regenerative potential of EVs derived from bone marrow MSCs (BMSCs) and adipose tissue MSCs (ADSCs) was compared. The EVs were isolated, characterized, and locally administered at the wound site.

In vitro, both types of EVs promoted the migration of FBs and KCs. However, in vivo, ADSC-derived EVs demonstrated significantly greater efficacy in promoting wound closure, accelerating re-epithelialization, enhancing angiogenesis, and boosting immunomodulation. miRNA and protein profiling analyses of the EVs revealed a partial overlap in molecular content between ADSC-EVs and BMSC-EVs. However, the presence of specific molecules selectively expressed in each EV type seems to explain the differences in their biological activities.

In particular, ADSC-EVs were enriched in pro-angiogenic miRNAs and proteins involved in key angiogenic signaling pathways, including Wnt, FGF, EGF, PDGF, and TGF. In contrast, BMSC-EVs were enriched in proteins related to cell adhesion and metabolic processes. In vivo studies confirmed that ADSC-EVs significantly accelerate the healing of diabetic wounds, while BMSC-EVs did not show significant therapeutic effects in the same model. Molecular analyses further confirmed the selective enrichment of key factors in ADSC-EVs, supporting their superior regenerative efficacy.

Exosomes secreted by macrophages modulate immune responses and promote inflammation resolution, thus contributing to diabetic wound healing and improving skin regeneration quality. This effect is due to their ability to accelerate both re-epithelialization and angiogenesis. Additionally, they attenuate inflammatory responses by reducing the secretion of TNF-α and IL-6, accelerating wound healing, reducing wound size, and promoting neovascularization, re-epithelialization, and the formation of granulation tissue rich in blood vessels [70].

Moreover, exosomes derived from platelet-rich plasma (PRP-EVs) have been shown to promote diabetic wound healing both in vitro and in vivo. Hyperglycemia induces oxidative stress and activates the JNK/p38 pathways, inhibiting fibroblast proliferation and survival. PRP-EVs counteract these effects by maintaining Bcl-2 expression and inhibiting Bax through JAK2/STAT3 pathway activation [71].

**Figure 1:**
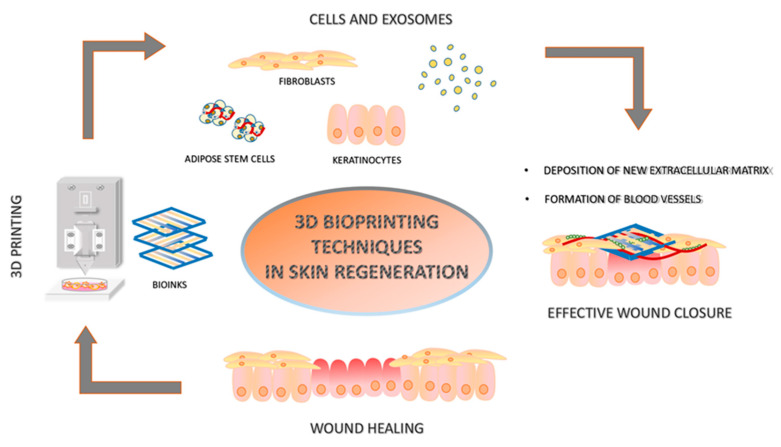
Three-dimensional bioprinting techniques in skin regeneration. At the bottom: an in vitro wound healing model. On the left: a 3D bioprinter together with bioinks that generate a scaffold. On top: cells and extracellular vesicles that can be inserted into or over the scaffold. On the right: the main effects of 3D-bioprinted application.

Recent advancements in 3D printing, coupled with the growing interest in therapeutic properties of EVs have led to numerous studies focused on integrating them into bioinks for the development of innovative scaffolds aimed at skin lesion regeneration. Due to their ability to modulate immune responses and their excellent biocompatibility, EVs are increasingly used as functional additives in bioinks to enhance the biological performance of bioprinting materials, addressing the limitations of conventional bioinks (Figure 1). Furthermore, their unique ability to evade the immune system and their compatibility with cells has made EVs a promising tool in 3D bioprinting, enabling their retention in the material and controlled release into the local target environment [72]. Three-dimensional-bioprinted exosomes have been used for bone repair, vascular engineering, neurodegenerative diseases, and skin injuries. In recent years, researchers have identified a relationship between exosomes and the physiological and pathological processes of the skin [73]. A study demonstrated the ability of sodium alginate (SA) and silk fibroin (SF) hydrogels to control EVs release. The freeze-dried MSC-secretome, called lyosecretome, was seeded at a concentration of 20 mg per ml SA-SF hydrogel. The results showed that the addition of SF to the hydrogel significantly delayed the release of lipids. SA-SF hydrogels control the release of EVs through a combination of diffusion and erosion [74].

In addition, a 3D hydrogel system called X-Block, which was fabricated using a mixture of biodegradable substrates, was generated. The porous network was distributed over 44% of the hydrogel volume. Moreover, continuous microchannels with diameters of 300 μm were dispersed inside the structure. Then, ADSCs were seeded at a density of 1.500 cells/cm^2^ showing an increased expression of several ‘‘stemness’’ associated markers, including LIF, OCT4, HGF, ZFP42, and NOTCH1 and the decrease in CASP3, a cell death marker. Moreover, ASCs cultured inside the tissue-mimetic system increased their protein secretion by approximately 50% and enhanced over two-fold extracellular vesicle (EV) secretion. The 100 kDa fraction of the 3D ASC secretome showed the ability to positively modulate human KC morphology towards the formation of a stratified cell layer with a spindle-like appearance, KC differentiation, metabolism, migration, and proliferation in in vitro assays [75]. Another study generated a bioactive EVM2-Gel ink by synthesizing a hydrogel from sodium alginate, calcium carbonate, and D-(+)-gluconic δ-lactone, to which extracellular vesicles derived from M2 macrophages (EVM2) were added. The resulting bioink was biocompatible and exhibited a degradation rate of 25% within 8 days, achieving complete degradation by day 12. The ‘portable bioactive ink for tissue healing’ (PAINT) technique was used. This technique uses a 3D printing pen that made the gel directly applicable to wounds of different shapes and sizes, adapted to specific geometries, and functioning as a portable biomedical device. In vivo experiments using a full-thickness 9 mm mouse circular wound model showed that EVs were continuously released into the wound site at high concentrations for a long time. Moreover, the bioink printed using PAINT technology was able to improve wound healing in a dose-dependent manner by inducing the switch of the macrophage phenotype from M1, pro-inflammatory, to M2, anti-inflammatory, and by promoting HUVECs proliferation and migration. In addition, there was high deposition of collagen fibers in EVM2-Gel treated mice [76].

Methacrylated hyaluronic acid (MeHA) bioink was used to fabricate 3D advanced smart patches loaded with exosomes derived from human mesenchymal stem cells (hMSC-EXOs). The scaffold had a parallelepiped shape with 50% pores that showed a square shape of approximately 700 μm. The 3D-printed MeHA patches were loaded with 200 μL of exosome suspension at a concentration of 4.8 mg/mL. After 16 h, MeHA patches released approximately 10% of their exosome content (0.451 ± 0.014 mg/mL), but their release reached a peak at 2 days (0.559 ± 0.045 mg/mL). In vitro wound healing assays on HDFs and HUVECs showed that cell proliferation and migration significantly increased after 24 and 48 h of stimulation with hMSC-EXOs released from the patch. Moreover, high expression levels of wound-healing-related COL1A1, COL3A1, and ELN mRNAs in FBs and CD31, kinase insert domain receptor (KDR), and von Willebrand factor (VWF) in endothelial cells have been demonstrated [77]. A summarized overview of EV-loaded bioinks is presented in Table 3.

## 5. Discussion

In adult mammals, the skin is a complex, multilayered organ. Following injury, the dermis lacks the ability to regenerate, and the epidermis can only restore in the presence of a dermal substrate, leading to scar tissue formation [78,79]. The most commonly used treatments for skin regeneration—including skin grafts, dermal substitutes, and wound sprays [17,23,25,26]—are associated with several limitations, such as the need for large donor areas, scar formation, graft rejection in the case of allotransplants [18,19,22], high costs, and immune responses [80]. In particular, the treatment of acute and chronic skin wounds is the main challenge in tissue regeneration. These wounds often result in significant pain and discomfort for patients and are frequently associated with metabolic disorders, vascular insufficiency, or mechanical trauma [81]. The healing process is impeded by several factors, including persistent tissue hypoxia, chronic inflammation, fibroblast senescence and dysfunction, imbalances in cytokines and growth factor signaling, dysregulated matrix metalloproteinase (MMP) activity, and microbial colonization that may progress to infection [82].

To overcome these drawbacks, 3D bioprinting has emerged as a promising and innovative alternative. This technique enables the precise deposition of living cells and biomaterials in complex geometries to fabricate functional human tissues and organs [83].

The formulation of a biocompatible and non-cytotoxic bioink is one of the most promising strategies in this context. Additionally, the incorporation of antimicrobial additives represents a new strategy, as bacterial infections are a major cause of complications during wound healing, making their management particularly complex [84].

The natural extract Satureja cuneifolia or the silver in biomaterials should be considered as a crucial strategy to minimize the risk of bacterial contamination, especially in hospital settings. Opportunistic pathogens such as *Staphylococcus aureus*, *Pseudomonas aeruginosa*, and *Escherichia coli*, which are commonly associated with nosocomial infections, pose a real threat to the success of advanced therapies [43]. Therefore, even in the absence of a direct regenerative effect, the integration of antimicrobial agents, whether natural or synthetic, could play a key role in maintaining wound site integrity, improving healing outcomes, and reducing the need for systemic antibiotic interventions.

All bioinks tested and discussed in this review have the potential to recreate structures mimicking native full-thickness skin. In particular, we highlight materials and techniques that could serve as innovative supports for promoting a more favorable microenvironment for skin regeneration and for developing a more faithfully layered equivalent skin model. The guanidinization of PEGylated chitosan [58], marine collagen from basa fish skin [60] or Co-2SS-GAM with HUVECs and HDFs [63] and mFAECM/GelMA/HAMA with HUVECs and HaCaTs [64] showed great potential for stimulating vascularized skin regeneration, because these 3D-printed materials are the most commonly used with a standardized protocol. The use of QHREDGS was tested for the first time in this study, demonstrating its ability to promote skin wound healing by prolonging peptide release in 3D-printed patches. Furthermore, GelMA/HAMA and QHREDGS contributed to efficient vascular reconstruction, with QHREDGS playing a key role in enhancing angiogenesis. Taken together, these results further suggest that 3D-printed peptide patches promote vessel formation and support wound repair in vivo. This innovative patch can be used for superficial wounds.

Among the various techniques, 3D prestress bioprinting could be useful for creating structures with a uniform and highly cellular arrangement, capable of stimulating cells to secrete pro-regenerative factors in a controlled manner. A stressed bioprinted filament mimics the physiological behavior of native tissue subjected to mechanical stress after damage.

The use of decellularized ECM (dECM) provides an innovative approach for the preparation of hydrogels with high biocompatibility and excellent properties, due to the presence of physiological components retained in the matrix, such as growth factors and cytokines. However, a key limitation remains the inability of dECM to be bioprinted without mixing it with other polymers owing to its low viscosity. Therefore, crosslinking with other polymers can significantly contribute to ECM bioprinting to achieve biomechanical properties that make it implantable as a scaffold. The ability to modulate immune response also makes the adECM-GelMA-HAMA scaffolds a promising candidate for further preclinical studies [65,66].

In the last part of the review, we summarized the most recent applications of EVs, particularly exosomes, into bioinks for 3D bioprinting as an innovative aspect. Their incorporation not only enhances tissue regeneration but also offers a ‘cell-free’ therapeutic strategy, reducing risks associated with cell transplantation, such as immune rejection and tumorigenesis [85]. The efficiency of EV encapsulation within bioinks largely depends on the bioink composition, as certain materials exhibit low retention capacity and uncontrolled release of EVs, compromising their therapeutic efficacy.

A notable example is the SA-SF hydrogel, which has been investigated as a controlled delivery system for EVs. The release profile was shown to depend on variables such as the treatment of silk fibroin and its molecular weight. Specifically, EVs release occurred more rapidly when the fibroin was degummed for one hour compared to longer treatments (2 or 4 h). This suggests that the density and organization of the polymer network significantly influence release dynamics, making these parameters critical for achieving effective control. However, this variability, combined with the lack of standardized protocols for fibroin preparation and for tuning the properties of the bioink, limits reproducibility and represents a major obstacle to large-scale clinical application [74], such as for EV isolation and storage.

In this context, the methacrylated hyaluronic acid (MeHA)-based bioink has shown promising results, when loaded with exosomes derived from human mesenchymal stem cells (hMSC-EXOs). MeHA enabled a homogeneous distribution of EVs within the 3D-printed structure and a controlled, sustained release for over seven days. The methacrylation of hyaluronic acid allows for the formation of a stable, highly biocompatible crosslinked network that effectively retains EVs without impairing their biological function. The released EVs maintained their ability to stimulate fibroblast proliferation and migration, as well as capillary-like structure formation in vitro [77].

The EVs were studied in another innovative 3D printing technique called PAINT that makes it possible to apply a gel to a damaged site. In addition, it would be interesting to evaluate the effects of the EVM2-gel ink on the macrophage population in preclinical studies, because the shift from M1 to M2 macrophages would quickly lead to the effective repair of damaged tissues [76].

All studies focus on the potential clinical use of 3D-printed materials with or without cells or EVs. However, to date, no clinical studies have been conducted in humans—only preclinical studies using animal models are available. Nowadays, case studies for human skin lesions were performed with only cells [86] or materials not bioprinted [87]. In particular, several clinical trials were conducted with MSCs [88], or with melanocyte and KCs, platelet-rich plasma and SVF [89]. Only two studies have been conducted on patients with burn injuries using 3D-printed silicone facemasks [90] or thermoplastic splinting material [91]. Preclinical studies on mice remain essential and elective models for studying skin wound healing for several reasons: ease of handling, rapid reproduction, and low cost compared to large animals such as rabbits or pigs. However, there are differences between humans and mice—wound healing in humans is primarily driven by re-epithelialization and granulation tissue formation, whereas in mice it is mainly the effect of contraction [92].

A noteworthy detail in this context is that 3D bioprinting, through the integration of advanced features such as biosensors, stimuli-responsive hydrogels, and controlled drug delivery systems, enables the fabrication of smart wound dressings capable of real-time monitoring and adaptation to the wound microenvironment. This active monitoring not only allows the creation of devices that closely mimic human skin, reducing patient discomfort, but also supports dynamic modulation of the healing process, leading to more efficient and personalized therapies [93]. Furthermore, the establishment of standardized production and printing protocols remains essential to ensure the reproducibility and reliability of results in view of potential clinical translation.

## 6. Conclusions

Skin regeneration is an important research topic in the scientific community. This review demonstrates the importance of the choice of bio-ink for both 3D bioprinting and cell biocompatibility. It provides several examples of advanced formulations that could soon be used in clinical practice, as well as innovative portable biomedical devices with the aim of immediately filling the lesion site with a bioprinted and biocompatible ink in a personalized manner.

## Figures and Tables

**Table 1:** Summary of acellular bioinks for skin regeneration.

Bioinks	Results	Reference
Gelatinmethacryloyl/hyaluronic acid methacryloyl (GelMA)/HAMA) added with glutamine-histidine-arginine-glutamic acid-aspartic acid-glycine-serine (QHREDGS) peptide	Full-thickness skin wound repair together with new vessels formation and ECM deposition in in vivo rat skin wound models.	[50]
5% placenta-derived extracellular matrix—sodium alginate (ECM-Alg)/Gel	Neo-angiogenesis in ex vivo CAM assay on chick embryos. Almost 87% of wound closure of full-thickness wound mice models together with new vascularization and collagen synthesis.	[51]
12% *w/v* xanthan gum/guar gum (XG)/GG)	Pro-angiogenic activity involves the formation of blood vessels and good tissue integration.	[52]

**Table 2:** Summary of cell-laden bioinks for skin regeneration.

Bioinks	Loaded Cells	Results	Reference
Gelatin Methacryloyl/methacrylated silk fibroin (GelMA)/SilMA)	Mice-derived FBs (Fibroblasts)	Full-thickness and vascularized skin wound repair together with ECM deposition in in vivo mice models.	[53]
Polyethylene oxide-co-Chitosan-co-polymethylmethacrylic-acid (PEO-CS-PMMA) added with Nicotinamide	Human dermal fibroblasts (HDFs)	Good biocompatibility for cells and good structural and mechanical properties for 3D bioprinting.	[54]
Gelatin and microbial transglutaminase (mTG)	Human skin FBs (HFF-1), mouse FB cells (L929), human bronchial epithelioid cells and mouse embryo osteoblast precursor cells (MC3T3)	In vivo full-thickness skin immunodeficient mice treated with stretched aligned HFF-1 laden scaffolds (SCLS) decreased wound dimensions. Moreover, a thick granulation tissue was generated, and the FBs inside it expressed higher levels of PCNA, demonstrating their high proliferation activity, and high VEGF levels, thus promoting angiogenic phenomena.	[55]
Gelatin Methacryloyl (GelMA) and a skin-derived extracellular matrix (SdECM)	Fibroblasts (FBs)	The hybrid GelMA/SdECM bioink exhibited higher viscosity compared to GelMA alone, improving printing stability. It also promoted greater fibroblast viability (97.6%) and proliferation, with increased expression of skin-related markers such as type I collagen and FGF, indicating enhanced biological functionality for skin tissue engineering.	[56]
Gelatin Methacryloyl—recombinant type III collagen (GelMA-rhCol3-3.2)	Human dermal fibroblasts (HDFs) and then human epidermal keratinocytes (HaCaTs) cells on top	Almost complete wound closure in full-thickness excisional wound rat models. Moreover, new hair follicles appeared, and collagen deposition was demonstrated.	[57]
Guanidinylated/PEGylated chitosan (GPCS)	Human dermal fibroblasts (HDFs) and then human epidermal keratinocytes (HaCaTs) cells on top	Generation of a thick skin with proliferating KCs layered on different planes in in vitro experiments.	[58]
Gelatin methacryloyl/hyaluronic acid methacryloyl (GelMA)/HAMA) added with 2 cm × 2 cm × 0.2 cm lattice cuboid	Normal human dermal FBs (NHDFs) and hair follicle dermal papilla cells (HFDPCs) spheroids. HaCaTs were seeded on the top	HaCaTs differentiated and penetrated into the bioprinted structure. Then, they surrounded HFDPC spheroids to form a complex in whom spontaneously hair-follicle-like unit (HFU) generated.	[59]
Semi-cross-linked alginate (ALG) mixed with 20 mg/mL marine collagen from basa fish skin (COL-ALG-20)	Normal human dermal fibroblasts (NHDFs) and human epidermal keratinocytes (HaCaTs)	NHDFs and HaCaTs proliferated inside the bioink and secreted new ECM.	[60]
Nanofibrillated Cellulose and Gellan Gum (NFC/GG)	Human epidermal keratinocytes (HaCaTs) cells	Very good cell survival after 7 days and very good rheological and mechanical properties for 3D bioprinting.	[61]
Sacrificial gelatin bioink	Human neonatal epidermal keratinocytes (HEKs)	HEKs adhered in the bioink and differentiated from the lower to the upper layer of the skin by forming a full-thickness skin model made by stratum basale, spinosum, granulosum, lucidum and corneum.	[62]
Cells in 2% Strontium silicate mixed with GAM (Co-2SS-GAM)	Human umbilical vascular endothelial cells (HUVECs) and human dermal fibroblasts (HDFs)	Thick epidermis regeneration with a dermal structure rich in collagen fibers and neo-vessels with the presence of hair follicles and complete epithelialization.	[63]
Microfragmented adipose extracellular matrix- Gelatin Methacryloyl- hyaluronic acid methacryloyl (mFAECM-GelMA-HAMA)	human epidermal keratinocytes (HaCaTs), Fibroblasts, human umbilical vascular endothelial cells (HUVECs)	Dermis regeneration in full-thickness excisional skin defect mice model by promoting the contraction of the new tissue inside the wound, a new vasculrization and collagen III deposition.	[64]
Human adipose tissue decellularized ECM-Gelatin Methacryloyl- hyaluronic acid methacryloyl (adECM-GelMA-HAMA)	Adipose-derived stem cells (ADSCs)	Complete wound closure together with collagen III deposition and neo-vessels formation in in vivo full-thickness injury mice model.	[65]
Human adipose tissue decellularized ECM-Gelatin Methacryloyl- hyaluronic acid methacryloyl (adECM-GelMA-HAMA)	Human adipose-derived stem cells (hADSCs)	Recovery of the entire area of wounded skin in full-thickness skin wound mice models, almost without scar tissue formation. Rich amount of collagen together with a modulated inflammatory cells response.	[66]
Decellularized and solubilized porcine skin tissue (S-dECM)	Human neonatal epidermal KCs (HEKs), endothelial progenitor cells (EPCs) and ASCs	Wound healing for over 21 days with the re-epithelialization and neovascularization phenomena, together with the enhancement of the blood flow in in vivo wound mice models.	[67]
3D PLC- enriched with 0.5% F-127 and immersed in a 0.5% gelatin solution	Bone-marrow-derived mesenchymal stem cells (BM-MSCs)	The developed material not only promotes granulation tissue formation, angiogenesis, and collagen deposition but also induces macrophage polarization toward the M2 phenotype, enhancing the expression of anti-inflammatory cytokines such as IL-4 and IL-10, which are essential for inflammation resolution and the healing process	[68]

**Table 3:** Summary of EV-loaded bioinks for skin regeneration.

Bioinks	Loaded Material	Results	Reference
Sodium alginate (SA) and silk fibroin (SF) hydrogels	Mesenchymal stem cell secretome (MSC-secretome)	Lyosecretome (freeze-dried MSC-secretome) was incorporated at 20 mg/mL into SA-SF hydrogels. The presence of SF significantly delayed lipid release, while EV release was modulated by both diffusion and matrix erosion.	[74]
X-Block hydrogel	Adipose stem cell secretome (ASC-secretome)	The ‘‘100 kDa’’ fraction of 3D ASC-secretome was able to positively modulate human KC (KCs) morphology towards the formation of a stratified cell layer, their differentiation, metabolism, migration and proliferation in in vitro assays.	[75]
EVM2-Gel ink	M2 macrophage-derived Evs (EVM2)	Improvement of wound healing in in vivo models, in a dose-dependent way, by inducing the switch of macrophage phenotype from M1 to M2 and by promoting HUVECs proliferation and migration. High secretion of collagen fibers.	[76]
MeHA	Exosomes derived from human mesenchymal stem cells (hMSC-EXOs)	Increased cell proliferation and migration after 24 and 48 h of stimulation with hMSC-EXOs released from the patch in in vitro assays.	[77]
